# Myeloperoxidase modulates human platelet aggregation via actin cytoskeleton reorganization and store-operated calcium entry

**DOI:** 10.1242/bio.20135314

**Published:** 2013-07-26

**Authors:** Irina V. Gorudko, Alexey V. Sokolov, Ekaterina V. Shamova, Natalia A. Grudinina, Elizaveta S. Drozd, Ludmila M. Shishlo, Daria V. Grigorieva, Sergey B. Bushuk, Boris A. Bushuk, Sergey A. Chizhik, Sergey N. Cherenkevich, Vadim B. Vasilyev, Oleg M. Panasenko

**Affiliations:** 1 Department of Biophysics, Belarusian State University, 220030 Minsk, Belarus; 2 Institute of Experimental Medicine, NW Branch of the Russian Academy of Medical Sciences, 197376 Saint-Petersburg, Russia; 3 A. V. Luikov Heat and Mass Transfer Institute of the National Academy of Sciences of Belarus, 220072 Minsk, Belarus; 4 N. N. Alexandrov National Cancer Center of Belarus, Lesnoy, 223040 Minsk, Belarus; 5 B. I. Stepanov Institute of Physics, National Academy of Science of Belarus, 220072 Minsk, Belarus; 6 Research Institute of Physico-Chemical Medicine, 119435 Moscow, Russia

**Keywords:** Myeloperoxidase, Platelets, Actin cytoskeleton, Aggregation, SOCE

## Abstract

Myeloperoxidase (MPO) is a heme-containing enzyme released from activated leukocytes into the extracellular space during inflammation. Its main function is the production of hypohalous acids that are potent oxidants. MPO can also modulate cell signaling and inflammatory responses independently of its enzymatic activity. Because MPO is regarded as an important risk factor for cardiovascular diseases associated with increased platelet activity, we studied the effects of MPO on human platelet functional properties. Laser scanning confocal microscopy was used to reveal carbohydrate-independent MPO binding to human platelet membrane. Adding MPO to platelets did not activate their aggregation under basal conditions (without agonist). In contrast, MPO augmented agonist-induced platelet aggregation, which was not prevented by MPO enzymatic activity inhibitors. It was found that exposure of platelets to MPO leads to actin cytoskeleton reorganization and an increase in their elasticity. Furthermore, MPO evoked a rise in cytosolic Ca^2+^ through enhancement of store-operated Ca^2+^ entry (SOCE). Together, these findings indicate that MPO is not a direct agonist but rather a mediator that binds to human platelets, induces actin cytoskeleton reorganization and affects the mechanical stiffness of human platelets, resulting in potentiating SOCE and agonist-induced human platelet aggregation. Therefore, an increased activity of platelets in vascular disease can, at least partly, be provided by MPO elevated concentrations.

## Introduction

Myeloperoxidase (MPO) is a heme-containing enzyme released from activated neutrophils and monocytes into the extracellular space and blood circulation during inflammation (Klebanoff, 2005). MPO catalyzes the formation of several reactive species, including hypohalous acids, that contribute to the destruction and killing of engulfed pathogens, and thus has a role in the innate immune response to foreign invasion. Besides hypohalous acids are involved in damage of biological macromolecules and tissue degradation in many diseases, especially those characterized by acute or chronic inflammation (Klebanoff, 2005).

MPO can also exert effects that are independent of its catalytic activity and affect various processes involved in cell signaling and cell–cell interactions, and are, as such, capable of modulating inflammatory responses. For example, MPO is able to bind to cellular membranes and modify some cellular functions. MPO can adhere to endothelial (Daphna et al., 1998) and epithelial cells (Haegens et al., 2008), fibroblasts (Zabucchi et al., 1989), bacteria (Miyasaki et al., 1987), macrophages (Lefkowitz et al., 1992) and neutrophils (Johansson et al., 1997). It seems likely that any cells located close to the activated and degranulated neutrophils can become targets for MPO.

In the past few years, evidences emerging from epidemiological studies have shown that higher concentrations of MPO are associated with an increased risk for cardiovascular disease (Baldus et al., 2003; Schindhelm et al., 2009), in the initiation and progression of which platelets are thought to play a predominant role (Vorchheimer and Becker, 2006). Platelets, also known as thrombocytes, are fragments of large bone marrow-derived cells called megakaryocytes. They have a biconvex disc structure with an equatorial diameter of 2–3 µm, and are akaryote. Their principal function is participation in blood coagulation by forming thrombi which are clots occluding the lumen of an injured vessel, thus stopping hemorrhage (Ruggeri, 2002). Recently, it was shown that platelets play the crucial role in regeneration of damaged tissues (Brass, 2010).

However, an increased activity of blood platelets can cause pathologic thrombus formation, which is the crucial event in pathogenesis of atherosclerosis, myocardial infarction, unstable stenocardia and other cardiovascular complications (Vorchheimer and Becker, 2006). There is evidence that platelet aggregation and MPO concentration in blood plasma increase concomitantly with progression of a cardiovascular disease (Pawlus et al., 2010), and numerous reports reveal the interrelations between platelets and neutrophils in inflammation and thrombosis (Siminiak et al., 1995; Khreiss et al., 2004). These and other observations raise a question whether MPO can bind to platelets, like it does with other cells, and if yes, whether their activity in the presence of MPO is changed.

Previous data obtained by indirect methods (peroxidase activity assayed by colorimetry of a chromogenic substrate in the presence of H_2_O_2_) suggested somewhat weak binding of MPO both to the blood platelets and neutrophils (Zabucchi et al., 1986; Zabucchi et al., 1989). However, recent studies using such sensitive methods as immunohistochemistry and flow cytometry, present the evidence of a specific binding of MPO to integrin CD11b/CD18 at the outer membrane of neutrophils followed by cell activation (Johansson et al., 1997; Lau et al., 2005). Yet, the question whether MPO affects platelets remained unanswered.

The present study was designed to ascertain MPO binding to human platelet membrane and to investigate effects of MPO on agonist-induced aggregation as well as on actin cytoskeleton reorganization, Ca^2+^ signaling and stiffness of these cells. We were able to demonstrate for the first time the role of MPO as mediator of human platelet activation.

## Materials and Methods

### Reagents and antibodies

Fura-2 acetoxymethyl ester (fura-2/AM) and BODIPY FL phallacidin were from Molecular Probes (Leiden, The Netherlands). Thrombin, HEPES, catalase, adenosine diphosphate (ADP), Triton X-100, thapsigargin (TG), fluorescein isothiocyanate (FITC)-conjugated mouse anti-rat IgG (anti-rat IgG FITC) antibody, sugars, poly-L-lysine and ionomycin (Iono) were from Sigma–Aldrich (St. Louis, MO, USA). Native MPO with RZ ∼0.85 was obtained from the donors' frozen leukocytic mass without using cationic detergents either during extraction or chromatographic purification of the enzyme (Sokolov et al., 2010). RZ (Reinheit Zahl) is used as a parameter of purity of myeloperoxidase and is defined as the ratio of absorbance at 430 and 280 nm (specific maximum of absorbance for hemin and aromatic amino acid groups, respectively). High-affinity polyclonal antibodies against MPO were prepared as described previously (Gorudko et al., 2009). All other reagents were of analytical grade.

### Platelet preparation

Venous blood samples were obtained from healthy donors at the Republican Scientific and Practical Center of Hematology and Transfusion (Minsk, Belarus). Blood was collected in tubes containing 3.8% trisodium citrate as anticoagulant at a ratio of 9:1. All procedures of human platelet isolation were conducted at room temperature, and use of glass containers and pipettes was avoided. Platelet-rich plasma (PRP) was obtained by blood centrifugation at 200 g for 10 minutes. Autologous platelet-poor plasma, obtained by further centrifugation at 600 g for 15 minutes, was used to adjust the platelet count of PRP to 2.5×10^8^ cells/ml. Washed platelets were prepared by additional two-step centrifugation of PRP at 600 g for 3 minutes, and the cell pellet was resuspended in Tris/ethylenediamine tetraacetic acid (EDTA) buffer solution (13.3 mM Tris, 120 mM NaCl, 15.4 mM KCl, 6 mM D-glucose, 1.5 mM EDTA, pH 6.9), with final cell concentration of 2.5×10^9^ cells/ml. This buffer prevented self-aggregation and clumping of stored platelets in our experiments.

### Analysis of MPO binding to human platelet membrane

Freshly washed platelets (2×10^8^ cells/ml) were incubated with gentle shaking in a phosphate buffered saline (PBS) (10 mM Na_2_HPO_4_/KH_2_PO_4_, 137 mM NaCl, 2.7 mM KCl, pH 7.4), containing 1 mM CaCl_2_ and 0.5 mM MgCl_2_ with or without MPO for 10 minutes at 37°C. In some experiments cell suspension was supplemented with sugars (α-methyl-D-mannoside, lactose or N-acetyl-D-glucosamine at the final concentrations of 60 mM) or either with 50% embryonic calf serum or platelet-poor plasma. Then cells were applied on poly-L-lysine-coated glass slides. After 30 minutes the samples were washed with PBS and fixed in 4% paraformaldehyde in PBS for 10 minutes, then again washed with PBS. To visualize MPO, cover slips with platelets were incubated for 1 hour in PBS containing anti-MPO antibody (1:5,000 dilution). After being washed with PBS three times to eliminate the excess of antibodies cover slips were kept for 1 hour in PBS containing anti-rat IgG-FITC antibodies (1:10,000 dilution). Then cells were again washed three times with PBS before mounting on slides. Images were acquired using laser scanning confocal microscope LSM 510 META (Carl Zeiss, Germany) with an immersion lens Plan Apochromat 63×/1.4 Oil (Carl Zeiss, Germany) and processed using LSM 510 software.

### Measurement of human platelet aggregation

Platelet aggregation studies were performed using both optical and impedance aggregometry. Platelet aggregation was detected by recording changes in light transmission at 540 nm of cell suspensions at 37°C on a computerized aggregometer AP 2110 from SOLAR (Minsk, Belarus). 400 µl PRP (2.5×10^8^ cells/ml) or washed platelets (2.5×10^8^ cells/ml) in PBS, containing 1 mM CaCl_2_ and 0.5 mM MgCl_2_ were incubated at 37°C with stirring for 3 minutes (with or without MPO) before adding an agonist (ADP to PRP and thrombin to washed platelets). Aggregation curves were recorded for 10 minutes. To quantify agonist-induced aggregation we used the maximal rate of cell aggregation calculated automatically by aggregometer software. Electronic impedance aggregation measurements were performed using an impedance lumi-aggregometer Chrono-log 700 (USA). An aliquot of whole blood (0.5 ml) was diluted with an equal volume of prewarmed isotonic saline and incubated for 5 minutes at 37°C with or without MPO. Impedance of each sample was monitored in sequential 1-minute intervals until a stable baseline was established. Then ADP (5 µM) was added and aggregation was continuously monitored. Impedance aggregometry results are expressed as amplitude (or maximum aggregation) [ohm] at 6 minutes after reagent addition.

### Determination of human platelet elasticity modulus based on atomic force microscopy (AFM) measurement

AFM measurements were performed as previously described (Shamova et al., 2011). Freshly washed platelets (2.5×10^8^ cells/ml) in PBS containing 1 mM CaCl_2_ and 0.5 mM MgCl_2_ were incubated with or without MPO for 5 minutes and fixed for 30 minutes in 1.5% glutaraldehyde in PBS. Then the cells were centrifuged for 3 minutes at 600 g. Cell pellet was washed twice with PBS and twice with distilled water. Obtained cells were applied on glass slides 10×10 mm and dried in air for several hours. All steps were performed at room temperature.

AFM observations were carried out using specialized experimental complex that combines functions of probe scanning (NT-206 microscope, MicroTestMachines, Belarus) and optical microscopy (Lempt optical system, Belarus). Cantilevers (NSC11) with spring constant of 0.03 N/m were used. Tip radius was checked by using a standard TGT01 silicon grating from NT-MDT (Moscow, Russia) and was 100 nm for Young's modulus determination. Measurements on ten separate cells were made in each specimen, and each cell was subjected to three penetrations of the probe into the cell. Young's modulus was calculated using Hertz's model as described previously (Chizhik et al., 1998) and used for estimation of platelet elasticity. In AFM experiments the probe indentation depth (25 nm) did not exceed 10% of a cell's height that allowed to neglect the influence of a rigid substrate on estimation of Young's modulus (Mathur et al., 2001).

### Measurement of intracellular free calcium concentration ([Ca^2+^]_i_)

Fluorescence was recorded in 2-ml aliquots of magnetically stirred cell suspensions at 37°C using fluorescent dye fura-2/AM. 9 µl of 0.5 mM fura-2/AM was added to 2 ml of PRP and the sample was left at room temperature for 45 minutes. Dye-loaded cells were washed from incubation medium by two-step centrifugation of PRP at 600 g for 3 minutes at room temperature, and the cell pellet was resuspended in HEPES buffer (10 mM HEPES, 145 mM NaCl, 10 mM D-glucose, 5 mM KCl, 1 mM MgCl_2_). The first washing was done at pH 6.9, the second one at pH 7.4.

The obtained platelets were stored as stock suspension of 2.5×10^9^ cells/ml. To measure [Ca^2+^]_i_ 1.1 ml of HEPES buffer and 100 µl of the stock suspension were mixed in a quartz cuvette of the LSF 1211A spectrofluorimeter (SOLAR, Belarus). Fluorescence spectra were recorded at 510 nm (excitation at 340 and 380 nm) at 37°C. Changes in [Ca^2+^]_i_ were monitored using the fura-2 340/380 fluorescence ratio and calibrated according to the method of Grynkiewicz et al. (Grynkiewicz et al., 1985). Store-operated Ca^2+^ entry (SOCE) was estimated using the integral of the rise in [Ca^2+^]_i_ for 2.5 minutes after addition of CaCl_2_ (Rosado et al., 2000). Ca^2+^ release were estimated using the integral of the rise in [Ca^2+^]_i_ for 5 minutes after addition of TG+Iono or ADP.

### Determination of actin cytoskeleton reorganization

Washed platelets (2.5×10^8^ cells/ml) in PBS containing 1 mM CaCl_2_ and 0.5 mM MgCl_2_ were incubated with or without MPO for 10 minutes at 37°C. Then cells were fixed in 4% paraformaldehyde in PBS for 10 minutes and centrifuged for 3 minutes at 600 g to wash away the fixing solution. Washed platelets were applied on cover slips and dried at room temperature for 10 minutes. Platelets were permeabilized for 5 minutes in PBS contained 0.5% Triton X-100 and washed three times with PBS. To visualize F-actin cells were stained with 0.07 µM BODIPY FL phallacidin in the dark for 45 minutes at room temperature and washed three times with PBS before mounting on slides.

Images were acquired using laser scanning confocal microscope (Carl Zeiss LSM 510 NLO, Germany) with an immersion lens C-Apochromat 40×/1.2 W (Carl Zeiss, Germany).

Statistical processing of experimental data was done to present mean values ± s.e.m. Significance of the differences among mean values was calculated using Student's test, considering those being significantly different in case of *P*<0.05.

## Results

### MPO binding to platelets

**Fig. 1.**
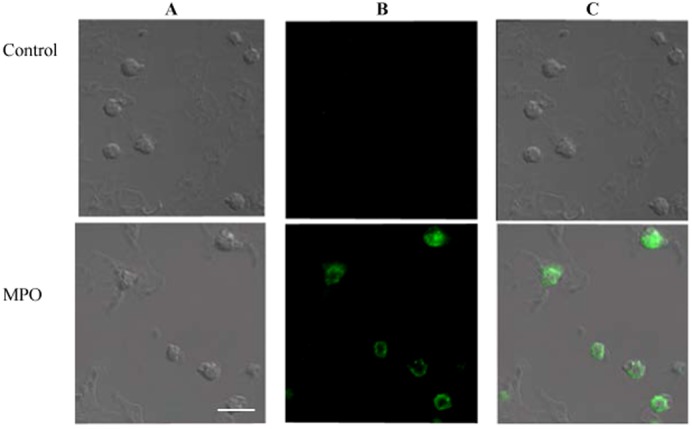
Confocal images showing MPO binding to platelet plasma membrane. Washed human platelets (2×10^7^ cells/ml) were incubated alone (upper section) or with MPO (100 nM) (lower section) for 10 minutes at 37°C, fixed with 4% paraformaldehyde and stained for MPO with an anti-MPO antibody and anti-rat IgG-FITC as described in Materials and Methods. (**A**) Transmission light microscopy. (**B**) Fluorescence microscopy of the same cells in panel A. (**C**) Merged images from panels A and B. Scale bar: 5 µm. The micrographs shown are representative of three separate experiments.

As laser scanning confocal microscopy allows direct visualization of molecule localization in cells, it was used to assay MPO binding to platelets. Washed platelets were incubated with MPO, after which high-affinity anti-MPO antibody and the second antibodies labelled with FITC were used to spot MPO-binding sites on cells. Fig. 1 (upper section) demonstrates that control platelets do not bind the second (labelled) antibodies and have no inherent fluorescence. In the presence of MPO (lower section) fluorescence is distributed over cell surface, which looks like a clearly delineated ring on the optical section and evidences the localization of MPO on the platelet plasma membrane. It should be noted that interaction of MPO with platelets did not depend on the presence in the medium of donors' plasma components, embryonic calf serum or such sugars as α-methyl-D-mannoside, lactose or N-acetyl-D-glucosamine at the final concentration of 60 mM (data not shown). Therefore, the results obtained indicate a specific interaction of MPO with platelet membrane.

### MPO potentiates platelet aggregation

To investigate whether platelet functions are changed as MPO binds to their surface, platelet aggregation studies were performed by both light transmission and impedance methods. Preliminary experiments showed that MPO (25–200 nM) did not induce platelet aggregation under basal conditions (without agonist).

**Fig. 2.**
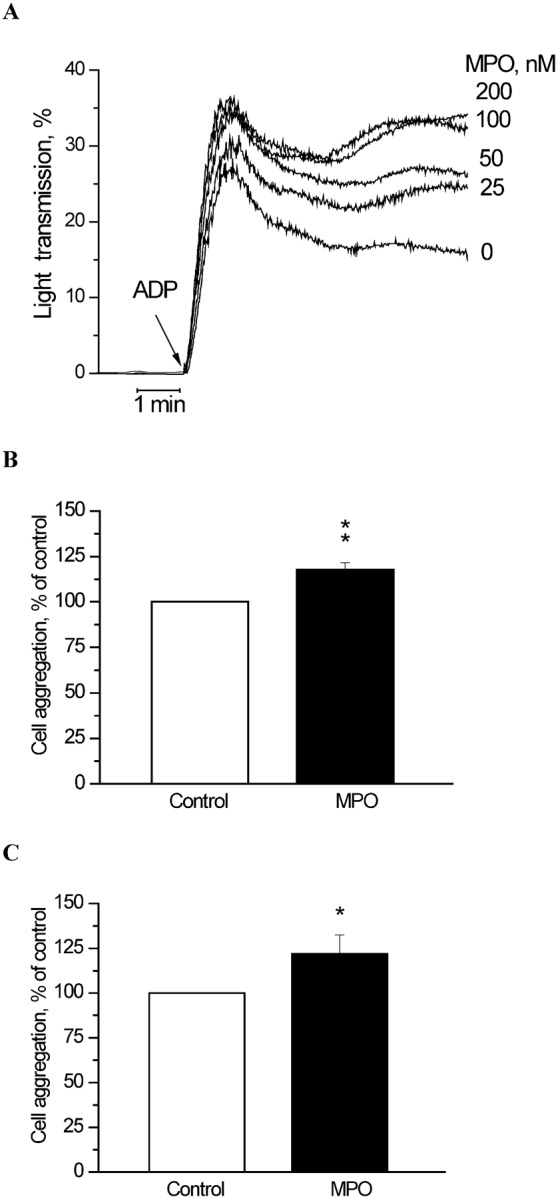
Effect of MPO on human platelet aggregation. (**A**) Typical kinetics of ADP-induced aggregation of PRP treated with different concentrations of MPO as specified on the curves (25–200 nM). ADP was used at concentration of 1 µM. Typical kinetics of three independent experiments are presented. (**B**) The rate of platelet aggregation activated by thrombin (48.4 mU/ml) in the presence of MPO (100 nM). (**C**) The extent of ADP-induced platelet aggregation (amplitude) in whole blood in the presence of MPO (100 nM). ADP was used at concentration of 5 µM. Each data point in panels B and C corresponds to mean ± s.e.m. of three independent experiments. *P<0.05; **P<0.01. In all cases platelets were treated with MPO for 2 min before adding the agonists.

Fig. 2A shows changes in light transmission of PRP induced by ADP. We used ADP at concentration of 1 µM, which provides a bi-phasic aggregation curve. A primary wave aggregation response occurs leading to internal platelet signal transduction that allows for the release of granule contents, which include fibrinogen, serotonin, ADP, Ca^2+^ and others, that potentiate the primary aggregation response and promote the secondary wave of aggregation (Jackson, 2007). As shown in Fig. 2A, MPO dose-dependently enhanced ADP-induced platelet aggregation in PRP. More pronounced effect of MPO on platelet aggregation was observed for secondary wave aggregation. MPO-dependent potentiation of ADP-induced platelet aggregation was not diminished in the presence of MPO enzymatic activity inhibitors (300 U/ml catalase or 50 µM 4-aminobensoic acid hydrazide) (data not shown).

MPO demonstrated similar potentiating effect on the thrombin-induced aggregation of washed platelets measured also by light transmission. In the presence of MPO (100 nM) the rate of platelet aggregation activated by thrombin (48.4 mU/ml) was increased by 17.8±0.2% (*P*<0.01; *n* = 3) (Fig. 2B).

Finally, the extent of ADP-induced platelet aggregation (amplitude) in whole blood incubated with MPO (100 nM) was 22.0±1.1% (*P*<0.05; *n* = 3) higher than the extent of platelet aggregation in blood without MPO (Fig. 2C).

### Effects of MPO on calcium signaling in human platelets

Human platelet activation depends on Ca^2+^ signaling that plays a key role in many essential cell processes, including secretion and platelet aggregation (Jackson et al., 2003; Jackson, 2007; Brass, 2010). The rise in free cytosolic Ca^2+^ that occurs during platelet activation is provided by two mechanisms: first, Ca^2+^ release from intracellular stores; second, Ca^2+^ entry via the channels of plasma membrane (Rink and Sage, 1990). An important mechanism for Ca^2+^ entry into platelets and other unexcitable cells is SOCE modulated by the filling state of Ca^2+^ stores. In this study we investigated the effect of MPO on ADP-induced release of Ca^2+^ from intracellular stores as well as on ADP-induced activation of SOCE. Besides, we studied the effect of MPO on SOCE resulting from depletion of Ca^2+^ intracellular stores caused by TG in combination with Iono. The former is a specific inhibitor of Ca^2+^-ATPase in cellular calcium stores, while the latter is a Ca^2+^-ionophore. Their combined effect results in complete Ca^2+^ release from the platelet intracellular stores (Rosado et al., 2004b).

**Fig. 3.**
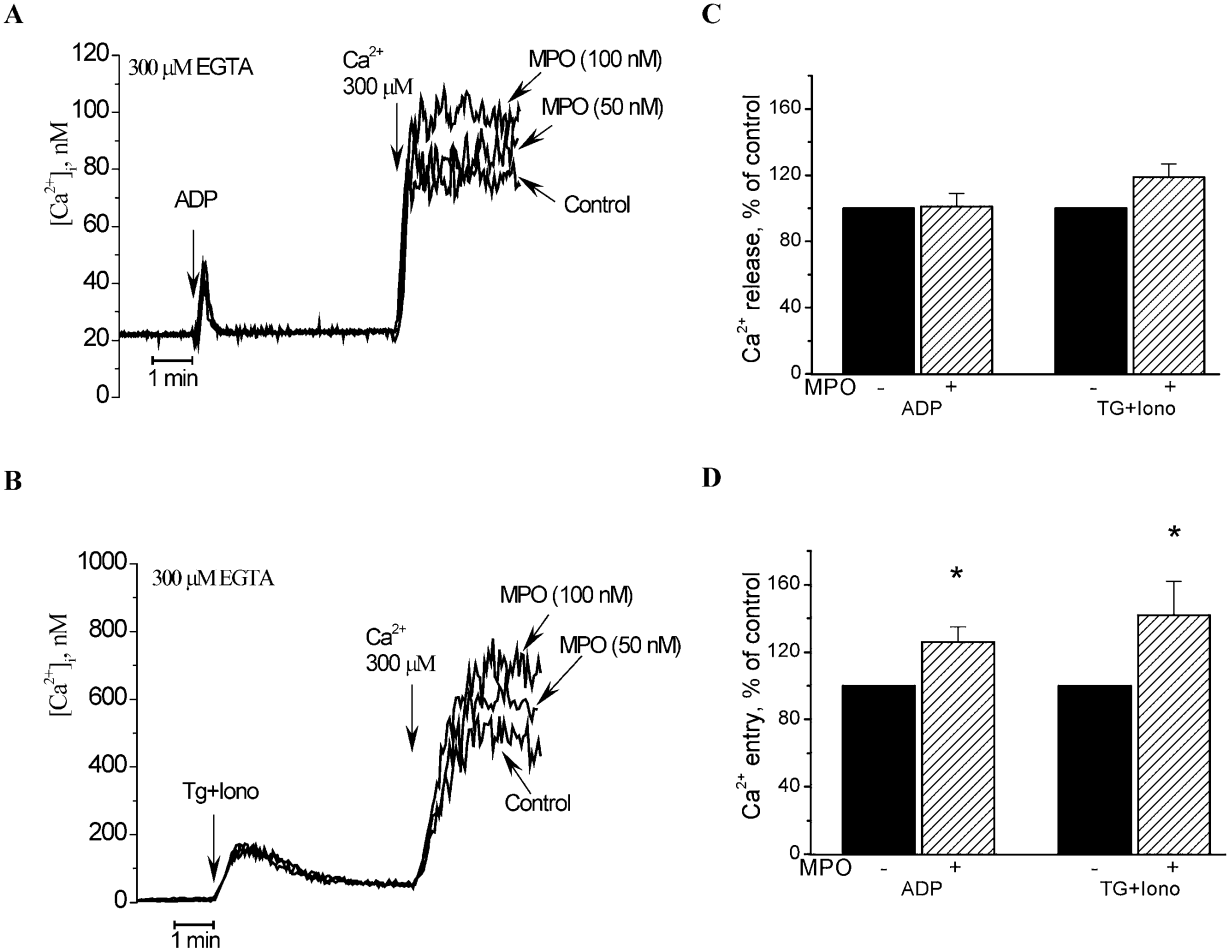
Effect of MPO on the activation of ADP- or TG+Iono-induced Ca^2+^ entry. (**A**) Fura-2-loaded platelets were incubated for 2 minutes at 37°C in the absence (control) or presence of different concentrations of MPO (50 and 100 nM) and then stimulated with ADP (1.3 µM) in a Ca^2+^-free medium (200 µM EGTA was added). 4 minutes later after addition of ADP CaCl_2_ (final concentration 300 µM) was then added to the medium of control or MPO-treated cells to initiate Ca^2+^ entry (activation of SOCE). Elevations in [Ca^2+^]_i_ were monitored using the 340/380 nm ratio, and traces were calibrated in terms of [Ca^2+^]_i_. (**B**) Fura-2-loaded platelets were incubated for 2 minutes at 37°C in presence of catalase (300 units/ml) without (control) and with different concentrations of MPO (50 and 100 nM) and then stimulated with TG (1 µM) plus Iono (50 nM) in a Ca^2+^-free medium (200 µM EGTA was added). 4 minutes later after addition of TG plus Iono CaCl_2_ (final concentration 300 µM) was then added to the medium of control or MPO-treated cells to initiate Ca^2+^ entry (activation of SOCE). Elevations in [Ca^2+^]_i_ were monitored using the 340/380 nm ratio, and traces were calibrated in terms of [Ca^2+^]_i_. (**C**,**D**) Histograms indicate the percentage of Ca^2+^ release and Ca^2+^ entry under the different experimental conditions relative to their control. Ca^2+^ entry was determined as described in Materials and Methods. Values are mean ± s.e.m. of three independent experiments. *P<0.05, *n* = 3.

As shown in Fig. 3A, treatment of platelets with ADP in a Ca^2+^-free medium induced increase in [Ca^2+^]_i_ due to Ca^2+^ release from intracellular stores. The subsequent addition of Ca^2+^ (300 µM) to the extracellular medium induces a prolonged elevation in [Ca^2+^]_i_ indicative of activation of SOCE. Treatment of platelets with MPO did not effect on Ca^2+^ release from intracellular stores but increased ADP-induced SOCE by 26±9% (*P*<0.05; *n* = 3) (Fig. 3C). These results suggest that cell exposure to MPO resulted in significantly enhanced SOCE induced by treatment with ADP (Fig. 3D).

Potentiating effect of MPO on SOCE in human platelets was confirmed in the experiments with TG and Iono. Since action of both agents on activation of SOCE in platelets is accompanied by production of hydrogen peroxide (Rosado et al., 2004b), which activates tyrosine kinase pp60*src* involved in the activation of SOCE, we investigated the effect of MPO on TG+Iono-induced SOCE in the presence of catalase (300 units/ml) (Rosado et al., 2004b): first, to reduce SOCE in order to study whether MPO can enhance Ca^2+^ entry; second, to inhibit the enzymatic activity of MPO. Adding MPO to platelets in the presence of catalase did not affect Ca^2+^ release from intracellular stores induced by TG(100 nM)+Iono (20 nM) (Fig. 3B,C), but significantly increased Ca^2+^ entry by 42±20% (*P*<0.05; *n* = 3) (Fig. 3B,C), indicative of a potentiating effect of MPO on SOCE. This finding further supports the notion that MPO enhances platelet activity independently of its enzymatic activity.

### MPO induces actin cytoskeleton reorganization in platelets

Actin cytoskeleton is likely to play an important role in the regulation of Ca^2+^ entry in platelets (Rosado et al., 2000; Rosado et al., 2004a; Rosado et al., 2004b). To test whether treatment of platelets with MPO induces actin cytoskeleton reorganization, human platelets were stimulated with MPO, fixed and filamentous actin was visualized with BIODIPY FL phallacidin.

**Fig. 4.**
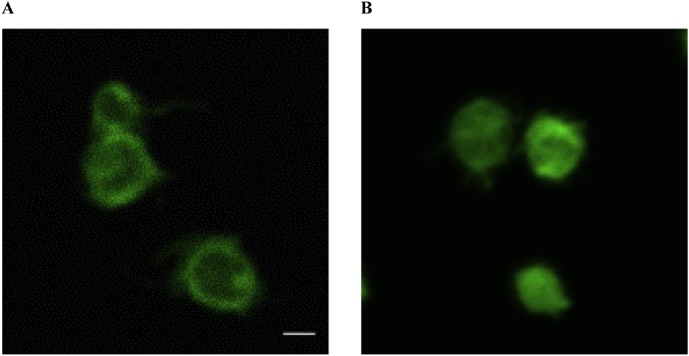
MPO induces actin cytoskeleton reorganization. Platelets were incubated at 37°C for 10 minutes without (**A**) or with (**B**) MPO (100 nM) and then fixed with 4% paraformaldehyde for 10 minutes at room temperature before further processing as described in Materials and Methods. Actin was visualized with BIODIPY FL phallacidin using a confocal microscope. Scale bar: 1 µm. The micrographs shown are representative of three separate experiments.

Fig. 4A shows typical distribution of F-actin in the resting platelets: actin filaments are located mainly at cell periphery as a submembrane skeleton and more of them diffuse in the cytosol. Treatment of platelets with MPO induced the peripheral redistribution of F-actin and an increase in F-actin throughout the cells (Fig. 4B). This result suggests that binding of MPO to platelets changes the distribution of their actin filaments resulting in depolymerization of near-membrane F-actin and polymerization of cytosolic actin network.

### MPO changes platelet mechanical properties

**Fig. 5.**
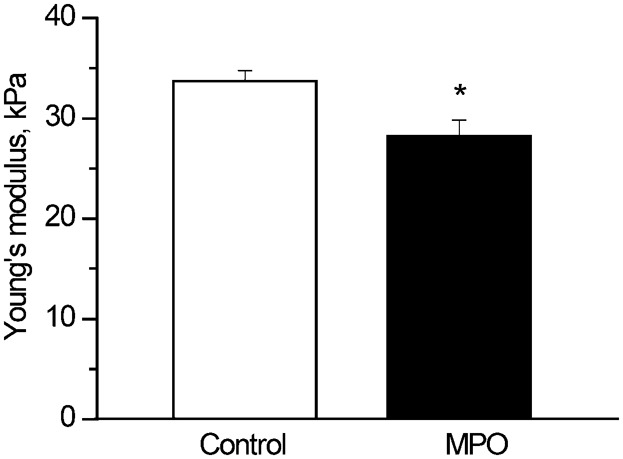
Effect of MPO on surface elasticity of human platelets. Platelets were incubated at 37°C for 10 minutes without (control) or with MPO (100 nM) and then fixed with 1.5% glutaraldehyde for 30 minutes at room temperature before further processing as described in Materials and Methods. Young's modulus of the platelets was measured in the central part of the cell, immediately after the sample preparation. The average values were calculated on the depth of penetration of 25 nm from the force-indentation curves using Hertz's model. Values are mean ± s.e.m. of three independent experiments. *P<0.05, *n* = 3.

Recent studies have reported that actin cytoskeleton reorganization affects mechanical features of cells (Cai et al., 2010; Kirmizis and Logothetidis, 2010). Elasticity is one of the most significant mechanical parameters of the cells. To investigate whether mechanical properties of platelets change in the presence of MPO we used AFM. As shown in Fig. 5, Young's modulus value for platelets treated with MPO was lower than that for unstimulated cells. It is indicated that platelets in the presence of MPO were less rigid than control cells. The results obtained by AFM are in line with the data obtained by laser confocal microscopy and taken together, they demonstrate that MPO induced platelet actin cytoskeleton reorganization and caused the elastic modulus decrease.

## Discussion

The present study provides novel insights into the role of MPO in inflammatory diseases. It is the first to report carbohydrate-independent MPO binding to human platelet membrane and reveal the role of MPO as a paracrine modulator of platelet function. The most important finding here is demonstration that MPO exerts potentiating effect on SOCE and agonist-induced platelet aggregation independently of its catalytic activity. We also have provided evidence that MPO induces alterations in the actin cytoskeleton and mechanical properties of human platelets. Since an increase of MPO level is a common feature of many pathological conditions, the ability of MPO to modulate platelet function may represent a mechanism enhancing thrombosis in inflammation.

Several studies have shown that MPO can adhere to endothelial cells (Vargunam et al., 1992; Daphna et al., 1998), leukocytes (Johansson et al., 1997), certain bacteria (Miyasaki et al., 1987) and yeast (Wright and Nelson, 1988), with retention of its activity. It is assumed that any cells that are close to activated neutrophils can be a potential target for MPO. Binding of MPO to cellular surface can change functional properties of cells through production of oxidants as well as independently of enzymatic activity of MPO. For instance, it was shown that MPO binding to microbial surfaces enhances the killing of *Actinobacillus actinomycetemcomitans* by the MPO-H_2_O_2_-Cl^−^ system (Miyasaki et al., 1987). MPO associated with bovine aorta endothelial cells inactivates factor IX-binding protein of endothelial cell surface abrogating the interaction of the binding protein with coagulation factor IX through involvement of MPO-generated hypochlorite (Daphna et al., 1998). On the other hand, binding of MPO to neutrophil CD11b/CD18 integrins stimulates neutrophil adhesion (Johansson et al., 1997), degranulation and superoxide production (Lau et al., 2005), and delayed apoptosis (El Kebir et al., 2008) through the activation of intracellular signaling pathways, independently of MPO catalytic activity.

Using both impedance and turbodimetrical aggregation assays we found that MPO enhanced agonist-induced platelet aggregation in PRP and whole blood as well as aggregation of isolated platelets. Accordingly, MPO is not a direct agonist, but rather a factor that potentiates platelet aggregation.

It is known that an elevation in the [Ca^2+^]_i_ through both the release of Ca^2+^ from intracellular stores and Ca^2+^ entry across plasma membrane plays a major role in platelet activation. One important route for Ca^2+^ entry, known as SOCE, is activated by depletion of the Ca^2+^ stores. In the present work we have tested the effects of MPO on Ca^2+^ signaling in platelets. Our results indicate that MPO had no effect on agonist-induced Ca^2+^ release from intracellular stores but increased SOCE in platelets. Although the precise mechanism by which the depletion of the intracellular Ca^2+^ stores leads to SOCE remain controversial several reports indicate the requirement for cytoskeleton changes (Rosado et al., 2000; Rosado et al., 2004a; Harper and Sage, 2007). It was shown that platelets possess two agonist-sensitive Ca^2+^ stores, the dense tubular system (DTS) and acidic Ca^2+^ stores (Rosado et al., 2004a; Zbidi et al., 2011) and discharge of these Ca^2+^ stores is sensed by STIM1 and STIM1, STIM2 respectively (Zbidi et al., 2011). Recent work suggested that SOCE is activated by association of store-operated channels on plasma membrane formed by Orai1, TRPC1 and TRPC6 with STIM proteins of DTS and acidic Ca^2+^ stores (Zbidi et al., 2011). These results are consistent with previous results of the same authors (Rosado et al., 2000; Rosado et al., 2004a) demonstrating that submembrane cortical actin network acts as a clamp that blocks interaction between DTS and acidic Ca^2+^ stores and plasma membrane and therefore reorganization of submembrane F-actin network permits the activation of Ca^2+^ entry. Furthermore, these authors demonstrated that SOCE controlled by depletion of DTS pools required new actin polymerization, probably to support membrane trafficking toward the plasma membrane (Rosado et al., 2004a). To further investigate the role of cytoskeleton changes in MPO-potentiated Ca^2+^ entry we used laser confocal fluorescence microscopy and showed that treating platelets with MPO induces reorganization of actin cytoskeleton in platelets and it seems likely that these cytoskeleton changes include both remodeling of submembrane cortical F actin cytoskeleton as well as an increase in F actin throughout the cells. This finding is in agreement with AFM platelet elasticity measurements that showed that MPO caused an increase in the platelets' elasticity. Thus, actin filaments have been reported to make the major contribution to cellular elasticity and their destabilization decreases the rigidity of cells (Cai et al., 2010; Kirmizis and Logothetidis, 2010).

The data presented here suggest that MPO-dependent increase in Ca^2+^ entry may be mediated by both redistribution of cortical actin cytoskeleton and increase of cytosolic actin network in platelets. However, the precise mechanisms by which MPO-induced cytoskeleton changes increase SOCE as well as the depletion of whichever type of intracellular Ca^2+^ stores is responsible for SOCE in the presence of MPO remain to be elucidated.

It should be noted that recently an important role in SOCE for platelet Na^+^/Ca^2+^ exchangers (NCXs) influenced by the actin cytoskeleton was demonstrated (Harper and Sage, 2007). It was shown that the NCX promotes dense granule secretion and that the autocoids released potentiated the initial Ca^2+^ signal due to SOC opening, with about 70% of the signal attributable to autocoid action (Harper et al., 2009). In the light of data presented here indicating role for MPO in potentiating SOCE and agonist-induced aggregation it will be important to determine whether MPO affects dense granule secretion. Further work will be required to verify the role of NCXs and autocrine signaling in MPO-potentiated Ca^2+^ entry.

**Fig. 6.**
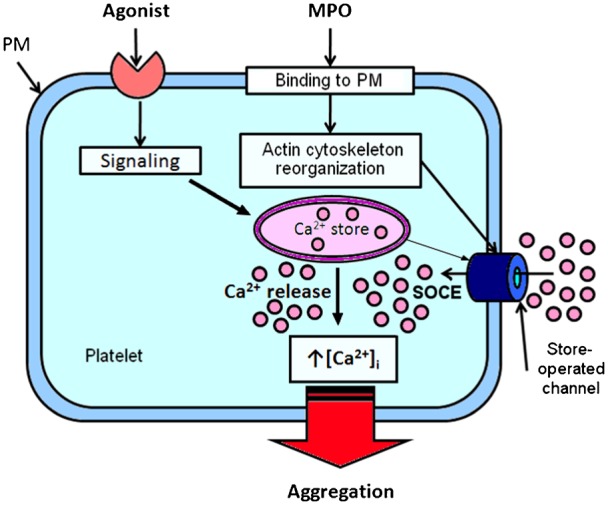
Schematic model depicting the role of MPO in regulating platelet activation. MPO binding to the platelet plasma membrane (PM) induces actin cytoskeleton reorganization that enables elevation in cytosolic Ca^2+^ concentration ([Ca^2+^]_i_) through enhancement of store-operated Ca^2+^ entry (SOCE) activated by depletion of intracellular Ca^2+^-store. An increase in [Ca^2+^]_i_ triggers Ca^2+^-dependent signaling processes leading to the potentiation of agonist-induced platelet aggregation.

Our findings and current understanding of MPO-mediated mechanisms in the activation of platelets are summarized in a schematic (Fig. 6), which is our working model for the future studies. Binding of MPO to platelet plasma membrane induces reorganization of the actin cytoskeleton and an elevation in the [Ca^2+^]_i_ through the potentiating SOCE. Ca^2+^-signaling triggers processes leading to potentiation of agonist-induced platelet aggregation and thrombus formation (or stabilization of platelet aggregates). It seems likely that Ca^2+^ being the major second messenger in cell signaling of platelets (Nesbitt et al., 2003) is the key regulator of MPO-mediated activity of platelets. It should be noted that potentiating effect of MPO on activation of SOCE as well as on agonist-induced platelet aggregation is not likely to be related to its enzymatic activity. These effects were observed in the presence of catalase that eliminated H_2_O_2_, the main substrate of MPO.

Molecular mechanism of MPO interaction with platelet surface remains unclear. It is known that MPO is a polycationic glycoprotein (pI>9) (Vargunam et al., 1992), consisting of 1.3% mannose, 0.6% glucose and 0.6% N-acetylglucosamine (Bakkenist et al., 1978). Interaction between MPO and cells may be of ionic nature (Vargunam et al., 1992; Klinke et al., 2011), carbohydrate-dependent (Miyasaki et al., 1987; Wright and Nelson, 1988) or receptor-mediated (Johansson et al., 1997; Lau et al., 2005). Some studies have shown that although mannan was able to inhibit binding of MPO to yeast and bacteria (Miyasaki et al., 1987; Wright and Nelson, 1988), it was unable to inhibit binding of MPO to leukocytes (Johansson et al., 1997; Lau et al., 2005). Consistent with this, we found that α-methyl-D-mannoside, lactose or N-acetyl-D-glucosamine did not inhibit the binding of MPO to platelets. This suggests that association of MPO with platelets as in the case with neutrophils is carbohydrate-independent.

MPO mediates neutrophil activation by association with CD11b/CD18 integrin (Johansson et al., 1997; Lau et al., 2005). This is an adhesion molecule which binds to a variety of other molecules, including fibrinogen, which is the principal ligand of the platelet glycoprotein IIb/IIIa (Rubel et al., 2001). On account of GP IIb–IIIa signals modulating cytoskeleton remodeling (Hartwig et al., 1999; Falet et al., 2005) and Ca^2+^-response of platelets (Bakkenist et al., 1978; Rubel et al., 2001; Klinke et al., 2011), it cannot be excluded that properly glycoprotein IIb/IIIa, playing the key role in formation of stable platelet aggregates, serves as a receptor for MPO.

Our evidence of platelet aggregation enhancement in the presence of MPO suggests that MPO can be a factor regulating the platelets' functions, providing a link between inflammation and formation of thrombi. Such a mechanism is likely to play an important role in the development of cardiovascular diseases. There is accumulating evidence that MPO displays potent proatherogenic properties (Klebanoff, 1999; Baldus et al., 2003). Immunohistochemical and biochemical analyses show localization of the enzyme and its oxidation products within human atherosclerotic lesions (Hazen and Heinecke, 1997; Sugiyama et al., 2001; Hazell et al., 2001; Thukkani et al., 2003). Suggested mechanisms through which MPO promotes inflammation and plaque formation include the production of hypohalous acids, which alter endothelial integrity and signaling by means of subendothelial matrix oxidation (Rees et al., 2012) as well as disrupt intracellular signaling (Klebanoff, 1999). MPO-mediated oxidation of high- and low-density lipoproteins promotes macrophage activation and foam cell formation (Podrez et al., 2000a; Malle et al., 2006). MPO-derived lipid oxidation products enriched in atheroma activate endothelial cells, promoting surface expression of P-selectin and activity of tissue factor, favoring platelet adhesion and coagulation cascade (Eiserich et al., 2002; Baldus et al., 2003). The ability of MPO to reduce NO bioavailability is responsible for endothelial disfunction and thrombogenic endothelial surface via expression of various prothrombotic and antifibrinolytic factors. Furthermore, MPO has been shown to activate metalloproteinases and promote destabilization and rupture of atherosclerotic plaque surface (Fu et al., 2001). In addition, MPO-triggered endothelial cell apoptosis has been suggested as a mechanism for the development of superficial erosions and a potential stimulus for platelet activation and aggregation (Ross, 1999; Podrez et al., 2000b; Heinecke, 2003). Revealed in this study the ability of MPO to increase the activity of platelets may serve as an additional mechanism for the involvement of MPO in the development of atherosclerosis. Further study will show whether platelets of patients with atherosclerotic lesions carry MPO on their surface. Such a notion is supported by the studies showing that different cells may bind MPO at sites of its high concentration (Lau et al., 2005; Li et al., 2007; Malle et al., 2000; Malle et al., 2006).

The results of the present study provide the first experimental evidence that MPO binds to human platelets, induces actin cytoskeleton reorganization and affects the mechanical stiffness of human platelets, resulting in potentiating SOCE and agonist-induced human platelet aggregation. Therefore, an increased activity of platelets in vascular disease at least partly can be provided by MPO elevated concentrations. Elucidation of molecular mechanisms responsible for MPO-dependent increase in platelet activity may be useful for the development of drugs intended to reduce the sensitivity of platelets to increased plasma concentrations of MPO.
